# Intradermal DNA vaccination combined with dual CTLA-4 and PD-1 blockade provides robust tumor immunity in murine melanoma

**DOI:** 10.1371/journal.pone.0217762

**Published:** 2019-05-31

**Authors:** Spela Kos, Alessandra Lopes, Veronique Preat, Maja Cemazar, Ursa Lampreht Tratar, Bernard Ucakar, Kevin Vanvarenberg, Gregor Sersa, Gaelle Vandermeulen

**Affiliations:** 1 Department of Experimental Oncology, Institute of Oncology Ljubljana, Ljubljana, Slovenia; 2 Advanced Drug Delivery and Biomaterials, Louvain Drug Research Institute, Université Catholique de Louvain, Brussels, Belgium; 3 Faculty of Health Sciences, University of Primorska, Izola, Slovenia; 4 Faculty of Health Sciences, University of Ljubljana, Ljubljana, Slovenia; Istituto Superiore di Sanità, ITALY

## Abstract

We aimed to explore whether the combination of intradermal DNA vaccination, to boost immune response against melanoma antigens, and immune checkpoint blockade, to alleviate immunosuppression, improves antitumor effectiveness in a murine B16F10 melanoma tumor model. Compared to single treatments, a combination of intradermal DNA vaccination (ovalbumin or gp100 plasmid adjuvanted with IL12 plasmid) and immune checkpoint CTLA-4/PD-1 blockade resulted in a significant delay in tumor growth and prolonged survival of treated mice. Strong activation of the immune response induced by combined treatment resulted in a significant antigen-specific immune response, with elevated production of antigen-specific IgG antibodies and increased intratumoral CD8^+^ infiltration. These results indicate a potential application of the combined DNA vaccination and immune checkpoint blockade, specifically, to enhance the efficacy of DNA vaccines and to overcome the resistance to immune checkpoint inhibitors in certain cancer types.

## Introduction

In recent years, the field of cancer immunotherapy has considerably expanded with several new treatment options [1]. Among them, DNA vaccines hold a great promise in prevention and treatment of different types of cancer. DNA vaccines are promising for cancer immunotherapy since they induce a broad immune response [2] with activation of both cellular and humoral arms of the adaptive immune system [3]. However, the clinical ability of DNA vaccines is still limited due to the poor immune response initially observed in humans. In order to increase the immunogenicity of DNA vaccines, novel improvements have been incorporated to the DNA vaccine platform, such as plasmid optimization, delivery by in vivo gene electrotransfer and use of genetically encoded immune adjuvants [4]. Gene electrotransfer is a well-established non-viral gene delivery method that has been used to deliver naked DNA or RNA to various tissues. Among them, gene electrotransfer of DNA vaccines into the skin has raised much attention, mainly due to the extended number of dendritic cells present in skin layers [5]. These cells are key players of the immune system able to orchestrate the activation and proliferation of T lymphocytes [6]. Skin appears thus as an ideal target for DNA vaccine administration and cutaneous gene electrotransfer of DNA has already demonstrated to be safe and efficient delivery technique, highly applicable to the clinical setting [7–9].

It is now clear that an effective immune response leading to significant antitumor effects requires not only an increase in immune activation but also reduction of suppressive or inhibitory elements of the immune system [10]. Therefore, in order to circumvent the lack of efficiency of DNA vaccines in humans and to overcome an immunosuppressive tumor microenvironment, there is a strong rationale for combining immune stimulating DNA vaccines with immune checkpoint inhibitors [10]. A number of antibody-based therapeutics targeting the immune checkpoint molecules have entered clinical trials and have been accepted by regulatory agencies [11,12]. Among them, immune checkpoint blockade with antibodies that target cytotoxic T lymphocyte-associated antigen 4 (CTLA-4) and the programmed cell death protein 1 pathway (PD-1/PD-L1) is demonstrating dramatic antitumor effects in subsets of patients in a variety of cancer types [13].

Despite the major success of immune checkpoint inhibitors, most patients still succumb to progressive disease, indicating that these therapies alone are insufficient to kill tumor cells completely [13]. Many cancer patients do not respond to treatment with immune checkpoint inhibitors, partly because of the lack of pre-existing tumor-infiltrating effector T cells [14]. This could be overcome with additional administration of cancer DNA vaccines that may prime patients for treatments with immune checkpoint inhibitors by inducing effector T-cell infiltration into the tumors and immune checkpoint signals. In this combination cancer DNA vaccines and immune checkpoint inhibitors may work hand in hand: cancer DNA vaccine-based immunotherapy may overcome the resistance of certain cancers to immune checkpoint inhibitors, while immune checkpoint inhibitors may reduce immunosuppression in the tumor microenvironment and enhance the efficiency of the cancer DNA vaccine therapies [14,15].

In previous studies, we established a safe and efficient gene electrotransfer protocol to deliver different DNA plasmids into skin, using a non-invasive multi-electrode array (MEA) for electric pulse application [16,17]. Here, based on an established method for skin delivery of DNA vaccines, we aimed to combine DNA vaccination method with immune checkpoint blockade and to elucidate the immune system involved in tumor recognition and elimination. The antitumor effect of combined treatment was evaluated in vivo in murine melanoma tumor model B16F10. To follow the immune responses involved in the antitumor activity in treated mice, we utilized a model pOVA DNA vaccine or a therapeutic vaccine coding for melanoma tumor antigen gp100 with a molecular adjuvant encoding murine IL12 in combination with antibodies that block immune checkpoint molecules CTLA-4 and PD-1. We hypothesized that dual CTLA-4/PD-1 blockade may increase the tumor immunity elicited by DNA vaccines and lead to a significant antitumor response in tumor-bearing mice compared to each treatment alone.

## Materials and methods

### Plasmids

The plasmids pVAX2-OVA, pVAX2-GP100 and pORF-mIL-12-ORT (here named pOVA, pGP100 and pIL12) were previously constructed [18–20]. All plasmids were isolated using the EndoFree Plasmid Mega or Giga Kit (Qiagen, Hilden, GE) according to manufacturer’s instructions and diluted in PBS. Plasmid DNA concentration and purity were assessed spectrophotometrically (260/280 ratio) and by agarose gel electrophoresis.

### Cell culture

Murine melanoma cells B16F10 (American Type Culture Collection—ATTC, Manassas, VA, USA) and B16F10-OVA (gift from Professor Johan Grooten, Ghent University, Belgium), a melanoma cell line from C57BL/6 mice that stably expresses ovalbumin, were cultured in minimum essential medium (MEM, Life Technologies, Carlsbad, CA, USA) supplemented with GlutaMAX with 10% fetal bovine serum (FBS), 100 μg/mL streptomycin, and 100 U/mL penicillin (Life Technologies) in a 5% CO_2_ humidified incubator at 37°C. Both cell lines were regularly checked and confirmed to be negative for *Mycoplasma* contamination.

### Animals

Female six- to eight-week-old C57BL/6 mice were obtained from Janvier (Le Genest-Saint-Isle, FR) or Envigo (Udine, IT). Mice were housed in pathogen-free conditions with 12-hour light cycles with *ad libitum* access to food and water. For tumor inoculation and electroporation, mice were anesthetized with 150 μL intraperitoneal injection containing a mixed solution of 10 mg/mL ketamine (Anesketin, euroVet, Heusden-Zolder, BE) and 1 mg/mL xylazine (Sigma, Diegem, BE) diluted in saline. All experimental protocols in mice were approved by the Ethical Committee for Animal Care and Use of medical Sector of the Université Catholique de Louvain (permission no. UCL/MD/2016/001) and the permission from the Veterinary Administration of the Ministry of Agriculture, Forestry and Food of the Republic of Slovenia (permission no. 34401-1/2015/16). The experimental procedures were performed in compliance with the guidelines for animal experiments of the EU directive (2010/63/EU).

### Tumor induction and tumor measurements

A total of 1 x 10^5^ B16F10-OVA or B16F10 cells diluted in 100 μL PBS were injected subcutaneously into the right flank of C57BL/6 mice. The day of tumor induction was set as day 0 in the experiments. Tumor size was measured three times per week with an electronic digital caliper. Tumor volume was calculated by a simplified formula as the length x width x height (in mm^3^) [3,19,20]. The results were presented with tumor growth curves or with Kaplan-Meier survival curves. Mice with B16F10-OVA tumors were sacrificed at day 16 after tumor induction, and spleen, blood and tumor samples were isolated for further analyses. In a case of the B16F10 tumor model, long-term effects on tumor growth and mice survival were followed up to 100 days after tumor induction. Mice were euthanized upon any sign of sickness or when tumor volume exceeded 1500 mm^3^. Mice with complete responses (tumor free for 100 days) were re-challenged with 1 x 10^5^ B16F10 tumor cells. The appearance of tumors in challenged mice was followed. To evaluate the additive effect of vaccination and immune checkpoint inhibitors on tumor growth, formula developed by Spector et al [21] for determination of the interaction between the two independent treatments was used.

### Animal immunization (intradermal electroporation)

Before immunization, the left flank was shaved using a rodent shaver (Aesculap Exacta shaver, AgnTho’s, Lidingö, SE). Thirty μL of PBS solution containing DNA plasmids was injected intradermally into left flank. Based on our previous studies, involving DNA vaccines and genetically encoded adjuvants [19,20], following concentrations of plasmid DNA were used: 50 μg of pVAX-gp100, 1 μg of highly immunogenic pVAX2-OVA and 1 μg of pORF-mIL-12-ORT, which was added as an adjuvant. Immediately after intradermal injection, electroporation was performed. Gene electrotransfer was performed under high voltage parameters (1600 V/cm, 100 μs) and MEA electrode (Iskra Medical, Podnart, SI) connected to Cliniporator (IGEA, Carpi, IT), was used to deliver 24 electric pulses. The exact procedure of gene electrotransfer and the composition of MEA electrode was described elsewhere [16,22]. For all the experiments, a conductive gel was used to ensure electrical contact with the skin (Aquasonic 100, Parker, Fairfield, CT, USA). The effect of electroporation alone and the effect of empty plasmids was described elsewhere [3,16,20].

### Injection of immune checkpoint inhibitors

To induce immune checkpoint blockade, the combination of two antibodies (BioXCell, West Lebanon, NH, USA) was used: anti-mouse PD-1 (αPD-1; clone 29F.1A12) and anti-mouse CTLA-4 (αCTLA-4; clone 9D9). Mice were given 100 μg of each antibody in a total volume of 200 μL delivered intraperitoneally, injected 3 times every third day (at day 3, 6 and 9).

### Determination of antigen-specific immune response

Mice in the experiment were previously immunized with DNA vaccine coding for OVA protein together with an adjuvant plasmid coding for IL-12 as described in *Animal immunization*. Tumors were excised 14 days after tumor induction and blood was collected aseptically for further determination of IgG titers. The wounds induced by tumor excision were stitched and target splenocytes were injected intravenously, as described in the *In vivo killing assay* section. At day 16 after tumor induction, mice were sacrificed and spleens were collected for further analysis. Before further analysis, the single cell suspension was prepared from spleen and tumor tissue. Red blood cells and spleen samples were lysed (ACK lysis buffer, Lonza, Walkersville, USA) and splenocytes were washed with PBS. Isolated tumors were incubated in media with 1 mg/mL Collagenase type II from *Clostridium hystoliticum* (Sigma) for 1 h and later washed with PBS and counted. In spleen tissue the percentage of antigen-specific killing was determined, in tumors the tetramer assay was performed and in serum samples the immunoglobulin titers were measured.

#### In vivo killing assay

Fourteen days after the immunization, mice in the experiment received a mix of target cells, prepared from naïve C57BL/6 splenocytes pulsed with the OVA H-2Kb-restricted epitope SIINFEKL (Anaspec Inc., Fremont, CA, USA) and fluorescently labeled with a high level of CFSE (eBioscience, San Diego, CA, USA) and of non-pulsed control cells labeled with a low level of CFSE. Briefly, splenocytes (2.5 x 10^6^ cells/mL PBS) from naïve mice were pulsed with 1 μg/mL of SIINFEKL in PBS for 1 h at 37 ⁰C. Pulsed splenocytes were stained with 5 μM (high) CFSE and non-pulsed controlled cells were stained 0.5 μM (low) CFSE. Two labeled cell populations were mixed at 1:1 ratio and 4 x 10^6^ cells of each population were adoptively transferred into each immunized mouse by intravenous injection in a total volume of 200 μL. Two days after the transfer, the spleen cells of recipient mice were isolated and stained with 100 μL of antibody mixture: Live/Dead-eFluor 506 (eBioscience) and Fc block (mCD16/CD32, BioLegend, San Diego, CA, USA) to reduce unspecific binding and α-F4/80 APC (BD Biosciences, San Diego, CA, USA) to exclude auto-fluorescent macrophages. Stained cells were incubated for 25 min at 4 ⁰C protected from light. Cells were analyzed by flow cytometry (FACSverse, BD Bioscience, Franklin Lakes, NJ, USA) to measure the amount of CFSE^high^ and CFSE^low^ cells. FITC positive cells and APC negative cells were included in further analysis. The percentage of antigen-specific killing was determined using the formula [20,23]: 100–100*((% CFSE^high^ cells/% CFSE^low^ cells)^immunized mice^/(% CFSE^high^ cells/% CFSE^low^ cells)^non-immunized mice^).

#### Tetramer assay

Tumors were excised and the single cell suspension was prepared from tumor tissue. Counted cells were stained with Live/Dead-eFluor 506 (eBioscience), Fc block (mCD16/CD32, BioLegend), CD3-APC-Cy7 (BioLegend), CD8a-FITC (BioLegend) and OVA-specific tetramers iTAg^TM^ MHC Tetramer H-2 Kb OVA (Tetramer-SIINFEKL-PE, MBL International Corporation, Woburn, MA, USA). According to the protocol (MBL International Corporation), stained cells were incubated at 4°C protected from light for a minimum of 1 h. Subsequently, flow cytometry (FACSverse) was performed to quantify the amount of antigen-specific T lymphocytes in tumor tissue. The amount of antigen-specific T cells was calculated using the formula: (% live cells * % single cells * % APC-Cy7^+^-PE^+^-FITC^+^ cells * counted cells) / tumor volume.

#### Immunoglobulin titers

An ELISA assay was performed to quantify the total immunoglobulin titers and IgG1 and IgG2 isotypes of anti-OVA antibodies in the serum samples. Briefly, 96-well plates were coated overnight with 10 μg/mL of OVA protein (Sigma) dissolved in sodium bicarbonate solution. After washing with 0.1% Tween20/PBS, plates were blocked for 30 min with 5% dry milk in PBS in a humid chamber at room temperature. After blocking, plates were washed and incubated for 1 h at room temperature with serial dilutions of serum samples diluted in 1% solution of bovine serum albumin (BSA) in PBS. Next, plates were washed and incubated for 1 h with the solution of following antibodies: peroxidase-labeled LO-MGCOC-2 (IMEX, University of Louvain, Brussels, BE) for determination of total immunoglobulin titers, LO-MG1-13 (IMEX) for determination of IgG1 titers and LO-MG2A-9 (IMEX) for IgG2a quantification. Final reaction was performed with 3,3’,5,5’–tetramethylbenzidine (TMB) substrate (Calbiochem, San Diego, CA, USA). Immunoglobulin titers were defined as the dilution factor giving an optical density at 450 nm equal to the limit of quantification (LOQ, mean blank value plus 10 SDs).

### Determination of the immunomudulating effect of combined treatment

Mice were previously immunized with DNA vaccine coding for gp100 together with an adjuvant plasmid coding for IL-12 as described in *Animal immunization*. Mice were sacrificed 16 days after tumor induction and the spleens and tumors were collected for further flow cytometry and immunohistochemistry.

#### Flow cytometry for determination of CD4, CD8, and FoxP3 positive T lymphocytes

Before flow cytometry analysis, splenocytes were lysed with ACK lysis buffer (Lonza) and washed with PBS. Isolated tumors were incubated in media with 1 mg/mL Collagenase type II from *Clostridium hystoliticum* (Sigma) for 1 h and later washed with PBS and counted. Prepared cells were stained with murine antibodies for 1 h at 4⁰C protected from light. The following antibodies were used in the study: Live/Dead-eFluor 506 (eBioscience), Fc block (mCD16/CD32, BioLegend), CD3-APC-Cy7 (BioLegend), CD8a-FITC (BioLegend), CD4-FITC (BioLegend), mFoxP3-APC (eBioscience). All data were collected on a FACSverse flow cytometer and analyzed using CaseViewer software (3DHISTECH Ltd., Budapest, HU).

#### Immunohistochemistry

Excised tumor and spleen tissues were fixed in 4% formaldehyde overnight and then cryopreserved in 30% sucrose for 24 h. Tissue samples were embedded in Tissue-Tek OCT (Sakura Finetek, Alphen aan den Rijn, NL) and sectioned at 8 μm using a cryostat (Leica Microsystems, Diegem, BE). Sections were stained with antibodies directed against murine CD4, CD8, and FoxP3. Briefly, sections were first blocked with 0.2% (v/v) Triton X-100 (Sigma), 10% (w/v) goat serum, 5% rat serum, and 2% BSA in PBS for 1 h at room temperature. Subsequently, the primary antibodies (rat CD8a-FITC 1:500 [clone 53–6.7, BioLegend] and CD4-FITC 1:500 [clone GK1.5, BioLegend] and mFoxP3-APC 1:500 [clone FJK-16s, eBioscience] were applied to the slides for 1 h at room temperature protected from the light. After washing with PBS, the sections were mounted using Vectashield Mounting Medium (Vector Laboratories, Burlingame, CA, USA) containing DAPI to visualize the cell nuclei. The slides were imaged using a structured illumination AxioImager microscope (Zeiss, Oberkochen, GE). The results are presented in *Supplemental Information*.

### Statistical analysis

For statistical analysis, Sigma Plot software (Systat software, London, United Kingdom) or GraphPad Prism 5 (GraphPad Software, San Diego, CA, USA) were used. Significance was determined by Student’s t-test or one-way analysis of variance (ANOVA) followed by a Holm-Sidak test, as recommended first-line procedure for pairwise comparison testing and also for comparison versus control. The analysis of survival after the tumor treatment was performed using the log-rank test. The P < 0.05 was considered significant. The values were expressed as arithmetic mean (AM) ± standard error of the mean (SEM).

## Results

### DNA vaccination in combination with CTLA-4/PD-1 blockade significantly delayed tumor growth in B16F10-OVA tumor-bearing mice

**Fig 1:**
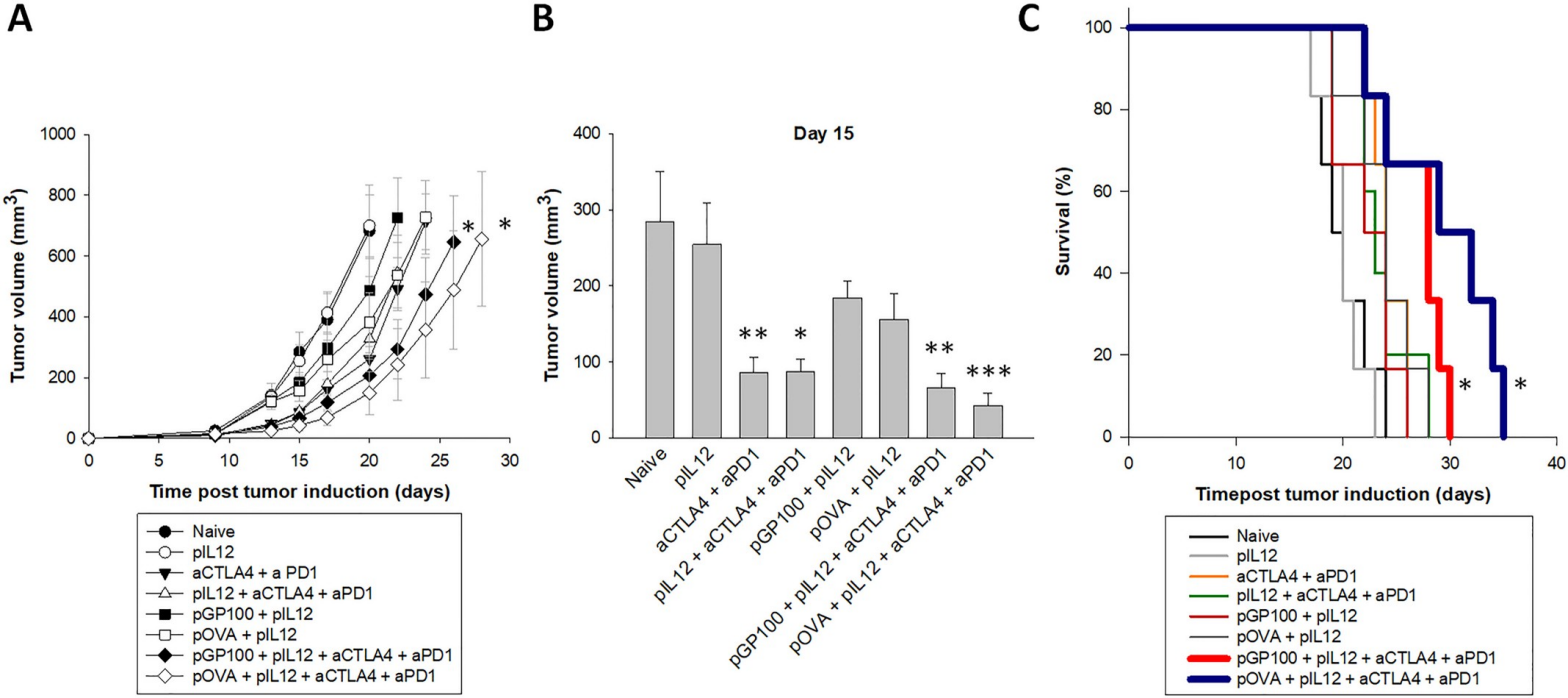
Tumor growth in B16F10-tumor bearing mice. **(A)** Tumor growth of B16F10-OVA tumors. Error bars indicate SEM. * statistically significant difference compared to the naïve group. **(B)** Tumor volume at day 15 post tumor induction. Statistically significant difference compared to naïve group: *(p<0.05), **(p<0.01), ***(p<0.001). **(C)** Kaplan-Meier survival curves of naïve and treated mice. * statistically significant difference compared to the naïve group. N = 6 mice per experimental group.

To test the research hypothesis that DNA vaccination and CTLA-4/PD-1 blockade may have complementary effects leading to a better control of tumor growth, mice were injected with B16F10-OVA tumor cells and received each individual treatment (plasmid encoding ovalbumin or gp100, pIL12 immune adjuvant and CTLA-4/PD-1 blockade antibodies) or a combination of them. Vaccination alone (pGP100 or pOVA), even with co-administration of the pIL12 molecular adjuvant, did not influence tumor growth in treated mice. At day 15, small tumor volumes were obtained in the groups treated with immune checkpoint inhibitors (αCTLA-4 + αPD-1) with or without the adjuvant plasmid coding for IL-12 and in the groups that combined DNA vaccination and inhibitors (pOVA + pIL12 + αCTLA-4 + αPD-1 and pGP100 + pIL12 + αCTLA-4 + αPD-1) (Fig 1A and 1B). However, only the treatments that combined an adjuvanted vaccine and the immune checkpoint inhibitors were able to significantly delay tumor growth of B16F10-OVA tumors compared to the naïve group (Fig 1A and 1C). The survival of mice that received an adjuvanted DNA vaccine (pOVA or pGP100) and CTLA-4/PD-1 blockade treatments was significantly prolonged as compared to naïve mice (Fig 1C). Mean survival times for pOVA + pIL12 + αCTLA-4 + αPD-1 group, pGP100 + pIL12 + αCTLA-4 + αPD-1 group and naïve group were 29, 27 and 20 days, respectively. Graph presenting the change of tumor volumes from the base line (spider plot) is included in S1 Fig. To highlight the respective contribution of DNA vaccination and immune checkpoint blockade, immunological analyses were performed using both ovalbumin as a model antigen and gp100 as a more relevant melanoma antigen.

### pOVA DNA vaccine in combination with CTLA-4/PD-1 blockade induced an antigen-specific immune response

**Fig 2:**
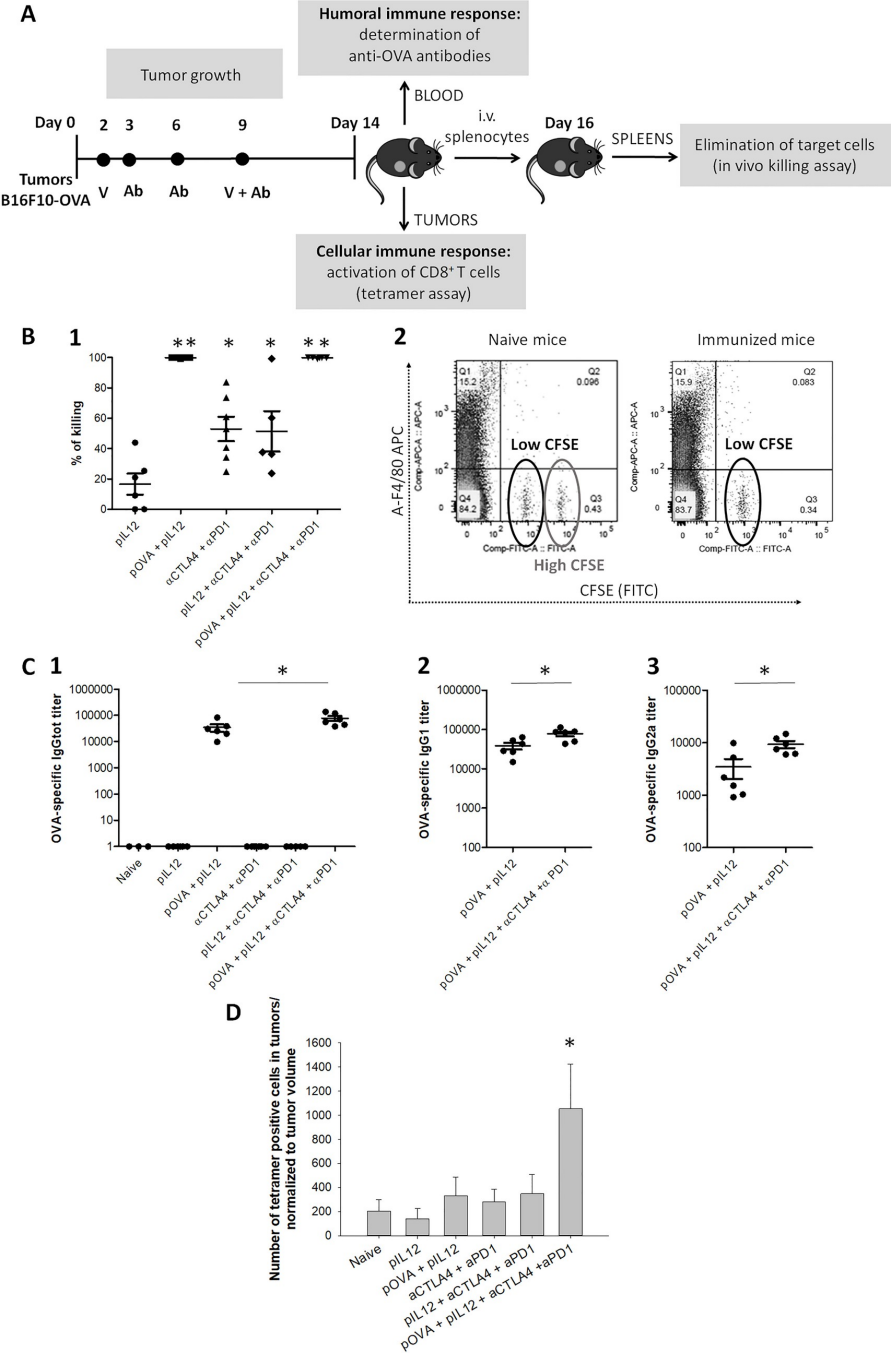
Antigen-specific immune response in B16F10-OVA tumor-bearing mice. **(A)** Time-line of the experiment with exact time points for the administration of DNA vaccine or immune checkpoint inhibitors. **(B)** In vivo killing assay; (1) Percentage of the killing of target cells. *statistically significant difference compared to the naïve group, ** statistically significant difference compared to all other groups. (2) Flow cytometry analysis presenting 2 separated peaks for low CFSE concentration and high CFSE concentration. In naïve mice, which were unable to eliminate target cells labeled with CFSE (FITC), both peaks are present. In mice immunized with pOVA vaccine, which completely eliminated target cells previously labeled with CFSE, the peak for high CFSE is absent. **(C)** IgG titers in serum samples; (1) Total anti-OVA IgG titer. (2) Anti-OVA IgG1 titer in immunized mice. (3) Anti-OVA IgG2A titer in immunized mice. *statistically significant difference between both marked experimental groups. **(D)** A number of tetramer-positive CD8^+^ T cells in tumors normalized to tumor volume. *statistically significant difference compared to the naïve group. Error bars indicate SEM. N = 7 mice per experimental group.

Activation of an antigen-specific immune response was followed in mice immunized with a DNA vaccine coding for OVA protein. To ensure the full potential of DNA vaccine, genetic adjuvant, *i*.*e*., a plasmid coding for IL-12 was co-delivered with DNA vaccine. DNA vaccination was further combined with CTLA-4 and PD-1 blockade. Cellular and humoral immune response against plasmid-encoded OVA antigen was examined. Blood and tumor were sampled 5 days after the end of the immunization schedule for determining anti-ovalbumin antibody titers and infiltrated specific CD8 T cells, respectively. Mice recovered well and were kept alive after tumor resection for two additional days. Their spleens were then collected to assess the systemic cellular response with an in vivo killing assay (Fig 2A).

#### DNA immunization resulted in complete elimination of target cells loaded with OVA antigen

The ability of mice to recognize and eliminate target cells was determined by an in vivo killing assay. At day 14 after tumor induction, target cells loaded with OVA peptide and labeled with CFSE were intravenously administered into naïve mice and mice treated with single or combined treatments. Two days later, the percentage of CFSE positive cells in excised spleens were quantified by flow cytometry. In both therapeutic groups immunized by intradermal gene electrotransfer of pOVA plasmid (pOVA + pIL12 and pOVA + pIL12 + αCTLA-4 + αPD-1 group), 100% of target cells were eliminated (Fig 2B, panel 1 and Fig 2B, panel 2). Complete elimination of target cells was observed in both immunized groups, whether treated with the vaccination alone or in combination with αCTLA-4 and αPD-1 antibodies. In the rest of the groups, which were not vaccinated with pOVA plasmid, the percentage of in vivo killing was significantly lower. These results indicate a strong antigen-specific systemic immune response induced by DNA vaccination. Complete elimination of target cells could be explained with higher production and activation of antigen-specific CD8^+^ cytotoxic T cells present systemically in treated mice. The OVA-specific immune response was further characterized by tetramer assay and measurements of IgG titers.

#### Antigen-specific IgG titers were significantly increased in immunized mice

To assess the activation of antigen-specific humoral immune response in treated mice, blood was collected at day 14 after tumor inoculation and anti-OVA IgG titers were determined in serum samples (Fig 2C). A strong humoral immune response against ovalbumin was obtained in mice immunized with pVAX2-OVA plasmid, demonstrated by highly increased total anti-OVA IgG titers (Fig 2C, panel 1). Total anti-OVA IgG titers, IgG1 (Fig 2C, panel 2) and IgG2a titers (Fig 2C, panel 3) were further increased when the DNA vaccination was combined with αCTLA-4 and αPD-1 antibodies. IgG1/IgG2a ratio did not statistically differ between both immunized groups. In mice which were not immunized the antigen-specific IgG antibodies were not detected. These results indicate that mice strongly responded to DNA vaccine by activation of B cells and production of antigen-specific IgG. The blockade of CTLA-4 and PD-1 combined with *DNA* vaccination generated a stronger humoral immune response with higher titers of OVA-specific IgG.

#### DNA vaccination in combination with CTLA-4/PD-1 blockade increased the intratumoral infiltration with antigen-specific CD8^+^ T cells

Tetramer assay was performed to quantify CD8^+^ T cells specific for given antigen (OVA) within a tumor tissue. At day 14 after tumor induction, tumors were excised and cells were stained with fluorescently labeled MHC-SIINFEKL tetramers. These tetramers specifically bind to T cell receptor on OVA-specific CD8^+^ T cells, what enables the quantification of tetramer-specific T cells in tumors. Between therapeutic groups, the amount of tetramer-positive antigen-specific CD8^+^ T cells in tumors was significantly increased only in the group treated with a combination of DNA vaccination with pOVA plasmid together with αCTLA-4 and αPD-1 antibodies (Fig 2D). In the rest of the groups, the number of tetramer-positive cells in tumors did not differ from the naïve group, indicating that DNA vaccine alone or inhibitors alone failed to induce the intratumoral infiltration of antigen-specific CD8^+^ T cells. By an in vivo killing assay, it was demonstrated that DNA vaccination promoted a systemic increase in CD8^+^ OVA-specific T cells. However, without the CTLA-4/PD-1 blockade, the CD8^+^ T cells did not reach the tumor where they need to act. These data suggest that the combination of DNA vaccination and immune checkpoint blockade is crucial for activation and infiltration of antigen-specific CD8^+^ locally at the tumor site, which is crucial for successful elimination of tumor cells. Similar results were obtained in our previously published study using DNA vaccine against murine P815 mastocytoma in combination with immune checkpoint blockade [24].

### Therapeutic immunization with pGP100 DNA vaccine promoted antitumor response when the DNA vaccine was combined with CTLA-4/PD-1 blockade

**Fig 3:**
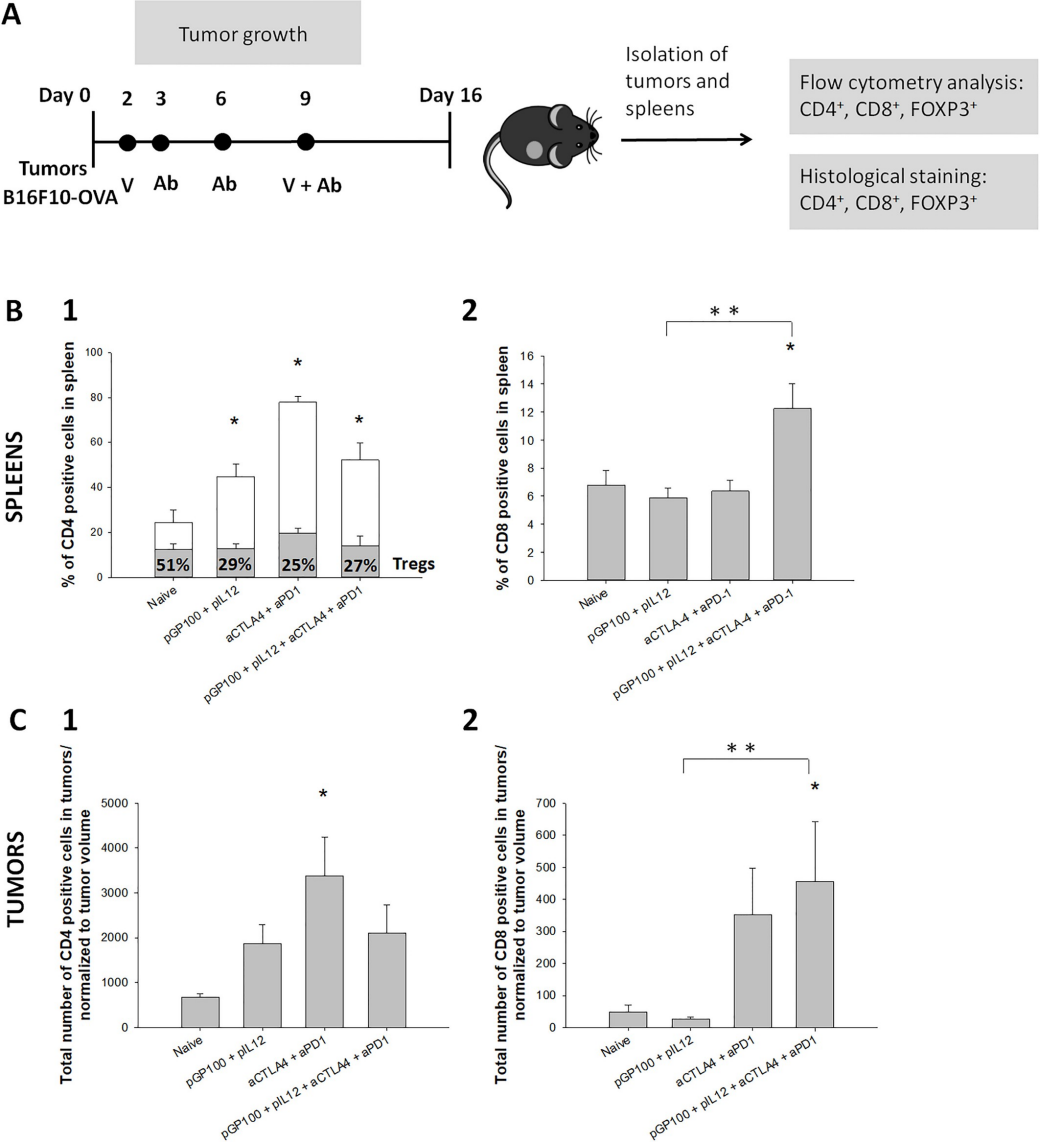
Effect of therapy combining therapeutic DNA vaccine coding for gp100 and dual CTLA-4/PD-1 blockade on the immune response. **(A)** Time-line of the experiment with exact time points for the administration of DNA vaccine or immune checkpoint inhibitors. **(B)** Infiltration of immune cells in spleen; (1) Percentage of CD4^+^ T cells in the spleen. CD4^+^ T cells were additionally divided to CD4^+^/FOXP3^-^ T helper cells (white column) and CD4^+^/FOXP3^+^ T regulatory cells (gray column with percentage marked with numbers inside the column). (2) Percentage of CD8^+^ T cells in the spleen. **(C)** Infiltration of immune cells in tumors. (1) A total number of CD4^+^ positive cells in tumors, normalized to tumor volume. (2) A total number of CD8^+^ positive cells in tumors, normalized to tumor volume. * statistically significant difference compared to the naïve group. ** statistically significant difference between combined group and vaccinated group. N = 8 mice per experimental group.

In subsequent experiments, instead of the plasmid coding for model antigen ovalbumin, a therapeutically relevant plasmid coding for melanoma antigen gp100 was used. A similar therapeutic protocol was used in order to follow the efficiency of combined therapy of DNA vaccine and CTLA-4/PD-1 immune checkpoint blockade. In subsequent experiments, the number of experimental groups was limited to main four groups, which demonstrated notable results after pOVA immunization. At day 16 after tumor induction, tumors and spleens of treated mice were excised. In excised tissue, CD4 and CD8 positive cells were determined (Fig 3A).

DNA vaccination alone and immune checkpoint inhibitors alone increased the percentage of CD4^+^ T helper cells, whereas, for induction of CD8^+^ T cells, the combination of both treatments is required. In spleens, the percentage of CD4^+^ T cells was significantly higher in all therapeutic groups compared to naïve group (Fig 3B, panel 1). With additional marker for FOXP3, CD4^+^ T cells were further divided to CD4^+^/FOXP3^+^ T regulatory cells (Tregs) and CD4^+^/FOXP3^-^ T helper cells (S2 Fig). In the naïve group, approximately half of the CD4^+^ cells were characterized as Tregs and half of them as T helper cells. DNA vaccine alone (pGP100 + pIL12), inhibitors alone (αCTLA-4/αPD-1) or combined treatment (pGP100 + pIL12 + αCTLA-4 + αPD-1) enhanced the percentage of CD4^+^ T helper cells in spleen up to 70%, with remaining 30% of Tregs. Percentage of CD8^+^ T cells in the spleen was significantly elevated only in the group treated with combined treatment of DNA vaccination and CTLA-4/PD-1 immune checkpoint blockade (Fig 3B, panel 2). In tumors, the number of CD4^+^ cells was higher in all therapeutic groups compared to naïve group (Fig 3C, panel 1), with the statistically significant difference in the group treated with immune checkpoint inhibitors alone. Among CD4^+^ T cells in tumors, up to 99% of them were characterized as T helper cells with remaining of less than 1% of Tregs in all experimental groups. Similar to results of measurements in spleens, the number of CD8^+^ T cells in tumors (Fig 3C, panel 2) was significantly increased only in the group treated with combined treatment (pGP100 + pIL12 + αCTLA-4 + αPD-1). Histology analysis (S3 and S4 Figs) confirmed the lower percentage of Tregs in spleens of treated mice together with higher CD8^+^ T cell infiltration in tumors of mice treated with combined therapy. The results indicate that treatment with pGP100 vaccine alone, treatment with CTLA-4/PD-1 immune checkpoint inhibitors alone as well as combined treatment with pGP100 vaccine and inhibitors contributed to the higher amount of CD4^+^ cells in tumors and spleens of treated mice with an increased percentage of immune stimulating T helper cells. Nevertheless, for the increased infiltration of effector T cells in tumors and spleens of treated mice, the combined treatment is required.

### Combined treatment of pGP100 DNA vaccine and CTLA-4/PD-1 blockade significantly delayed tumor growth and improved the survival of mice with B16F10 tumors

**Fig 4:**
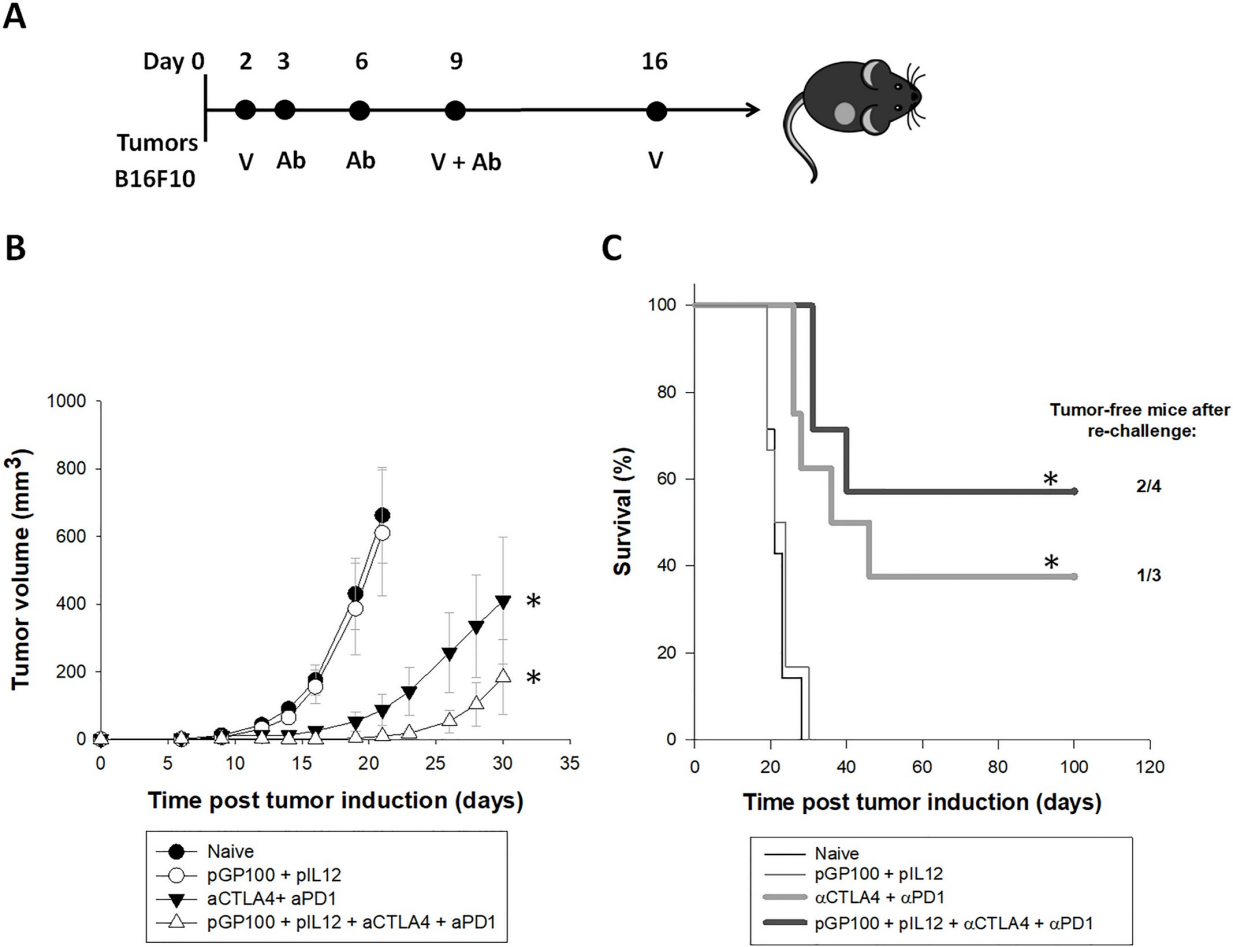
Long-term antitumor effect of combined treatment in B16F10 tumor-bearing mice. **(A)** Time-line of the experiment with exact time points for the administration of DNA vaccine or immune checkpoint inhibitors. **(B)** Tumor growth of B16F10 tumors. Error bars indicate SEM. * statistically significant difference compared to the naïve group. **(C)** Kaplan-Meier survival curves of mice after tumor challenge. * statistically significant difference compared to the naïve group. n.s. = no statistically significant difference. N = 8 mice per experimental group.

The long-term effect of pGP100 DNA vaccine and CTLA-4/PD-1 immune checkpoint blockade on tumor growth and mice survival was followed in naïve and treated mice bearing B16F10 tumors (Fig 4A). Similar to observations in B16F10-OVA tumor model, DNA vaccination alone was insufficient in delaying tumor growth in B16F10 tumor-bearing mice (Fig 4B). On other hand, CTLA-4/PD-1 inhibitors alone or in the combination with DNA vaccine efficiently delayed tumor growth and significantly prolonged mouse survival. Additionally, the formula developed by Spector et al [21] for determination of the interaction between the two independent treatments was used. The calculation confirmed the additive effect of DNA vaccination and immune checkpoint inhibitors, resulting in the prolonged tumor growth when both treatments were combined. In the group treated with pGP100 vaccine in combination with the CTLA-4/PD-1 blockade, ~60% of mice responded completely to the treatment, as opposed to ~40% of mice in the group treated with inhibitors alone (Fig 4C). The formation of immunological memory in the cured mice was determined by the secondary challenge of mice with B16F10 tumor cells. Tumor free-survival after re-challenge was detected in half of the mice treated with combined therapy and in one-third of mice treated with CTLA-4/PD-1 immune checkpoint inhibitors alone (Fig 4C).

## Discussion

The combined treatment of intradermal DNA vaccination and dual CTLA-4 and PD-1 blockade generated more robust antitumor activity compared to each treatment alone. It is now clear that different tumor types differentially respond to immunotherapy [25]. In this study performed with an aggressive murine melanoma model, we observed at day 15 that tumors were smaller in mice treated with αCTLA-4/αPD-1 antibodies alone compared to the naïve group. However, at later time-points, tumors were growing and the survival of mice was finally not improved by this single treatment. In addition, DNA vaccination alone failed to reject B16F10-OVA tumors although immunized mice strongly responded against OVA antigen with complete killing of OVA SIINFEKL peptide-loaded target cells and high production of detected anti-OVA IgG antibodies. Compared to single treatments, combined treatments resulted in increased intratumoral CD8^+^ T cells, what seems to be a crucial factor for successful tumor elimination based on present experiments and literature [26]. Indeed, the combination of pOVA DNA vaccine and immune checkpoint inhibitors induced strong activation of antigen-specific immune response. In B16F10-OVA tumor-bearing mice, this was demonstrated by the complete elimination of target cells pulsed with OVA antigen together with significantly increased OVA-specific cytotoxic CD8^+^ T cells in tumors and increased production of anti-OVA antibodies in serum. Similarly, combined treatment with pGP100 DNA vaccine and immune checkpoint blockade contributed to a higher percentage of CD4^+^ T helper cells in spleen together with increased infiltration of cytotoxic CD8^+^ T cells in tumors. Each treatment alone failed to induce the intratumoral infiltration of cytotoxic lymphocyte T, indicating that DNA vaccination or antibody therapies alone are insufficient to promote complete tumor cell killing. In contrast, strong activation of the immune response induced by combined treatment resulted in significant tumor growth delay and prolonged survival of the treated mice with a high level of complete responses. Half of the mice in combined group completely rejected a secondary B16F10 tumor re-challenge, indicating that the mice treated with DNA vaccine together with immune checkpoint blockade developed memory T cells and established long-term immunological memory to tumor antigens expressed in B16F10 tumors [27,28].

Despite promising results in preclinical models, the low immunogenicity of DNA vaccines and poor response to immune checkpoint blockade in many patients limit their use in clinical settings. In the scope of this study, we explore different steps towards the better antitumor activity of both therapeutic approaches, such as co-administration of genetic adjuvants, dual immune checkpoint blockade and combined treatment of DNA vaccination and antibodies targeting CTLA-4 and PD-1.

First, the genetic adjuvant, *i*.*e*., plasmid coding for IL-12, was co-delivered with DNA vaccines. In our previous studies, it was demonstrated that the immunogenicity of the antigens gp100 and OVA could be enhanced, if genetically encoded adjuvant pGAG was co-delivered with DNA vaccines [20]. Instead of pGAG, here we administered pIL12, a well-known immunomodulatory cytokine with antitumor activity [29], which generates local and systemic immune response. As a therapeutic agent, IL-12 has already entered clinical trials for treatment of metastatic melanoma [30,31]. Here, a plasmid coding for IL-12 administered in low concentration was considered as a clinically established genetic adjuvant, which may importantly contribute to the better applicability of DNA vaccine. The efficiency of IL-12 as a genetic adjuvant was evaluated and confirmed in many preclinical studies in different animal models [32,33]. The effect of more potent vaccines, such as DNA vaccines with multiple tumor antigens, needs to be further evaluated.

Second, the antitumor activity of immune checkpoint blockade may be enhanced, if antibodies targeting different immune checkpoint molecules are combined. There is clear rational to combine anti-CTLA-4 and PD-1 inhibitors, since both molecules act by different mechanisms to the impaired immune system. Since dual blockade of CTLA-4 and PD-1 in both animal models and in the clinic has shown improved antitumor responses compared to treatment with each checkpoint alone [34–37], the same combination was applied and explored in the present study.

Third, it is now clear that effective anticancer response could be achieved, if the balance between immune activation and reduction of suppressive elements of the immune system is established [10,37]. Thus, the drawbacks of DNA vaccination or inhibitors alone may be overcome by combined therapy of both treatment approaches [10,14]. The combination of cancer vaccines and immune checkpoint blockade was thoroughly studied in the case of cell-based vaccines [38–40]. In cell-based vaccine studies in which triple-combination therapies were used (cancer vaccine GVAX/FVAX together with αCTLA4/αPD-1 antibodies), the triple combination was profoundly superior to any of the double- or mono-therapies [11,38,41]. The strong antitumor effect observed after combined treatment with cell-based vaccines and inhibitors was ascribed to the increased proliferation of antigen-specific effector CD8^+^ and CD4^+^ T cells, antigen-specific cytokine release, inhibition of suppressive functions of Tregs and upregulation of key signaling molecules critical for T cell function [38]. On contrary, only a handful of preclinical studies have examined the efficacy of combining DNA vaccines with immune checkpoint inhibitors [11]. Published studies explored the combination of single or dual CTLA4/PD-1 blockade together with DNA vaccines targeting the MYB oncoprotein for colorectal cancer [42], SSX2 cancer antigen for sarcoma [43], PSMA prostate-specific antigen, P815 mastocytoma [24] and TRP-2 and gp100 melanoma-specific antigens [44]. The common thread of these studies is a promising anticancer effect achieved by combination therapy compared to each treatment alone [24]. However, it is difficult to compare and evaluate our results with regard to the results of the published studies since the selected tumor models, DNA delivery techniques, the immunogenicity of DNA vaccines, selected target tissues for DNA delivery and the treatment protocols greatly differ between the studies.

In a recent study involving gene electrotransfer of DNA vaccine targeting TERT in combination with the CTLA4/PD-1 blockade in TC-1 tumor model [11], it was reported that blockade of CTLA-4 and PD-1 synergized with TERT vaccine, generating stronger antitumor activity compared to checkpoint alone or vaccine alone. Immune checkpoint blockade was responsible for an enhanced percentage of CD8^+^ T cells and decreased the percentage of Tregs within the tumor. Despite the strong antitumor synergy, none of these immune checkpoint therapies showed improved TERT antigen-specific immune response in tumor-bearing mice. In contrast, in our study, the combined treatment with the dual blockade and electroporation-based DNA vaccine significantly promoted antigen-specific response with higher activation of antigen-specific CD8^+^ T cells and production of antigen-specific antibodies. Although T cells are key players in tumor elimination and present main targets for immune checkpoint inhibitors, special attention should be given in future studies also to the B cells involved in the tumor immunity [45]. In contrast, to study involving DNA vaccination targeting TERT, our combined treatment increased the percentage of Tregs in the spleen, but not within the tumors. These data suggest that immune checkpoint blockade functions to alter the immune microenvironment and expand effector T cells at the tumor site rather than acting on induction of Tregs.

Aside from intramuscular delivery of DNA vaccine, skin is a promising target for gene therapy and DNA vaccination [46]. Due to its easy accessibility, large treatment area, and the presence of many antigen-presenting cells, the skin is an attractive target particularly for delivery of DNA plasmids encoding different tumor antigens. In previous studies, we established safe and efficient gene delivery protocol to deliver different plasmids into skin, using MEA electrode for electric pulse application [16,17,22]. Gene electrotransfer with MEA electrode is a non-invasive procedure for skin treatment, which can greatly reduce the adverse effects of needle or plate electrodes [47–50]. Electroporation with MEA electrode induces minimal discomfort and is applicable particularly when multiple treatments are required, such as multiple administrations of DNA vaccines. The use of such electrodes is thus warranted in further studies and may greatly contribute to the clinical availability of electroporation-based DNA vaccination.

To conclude, the novelty of our study is the combination of gp100 or OVA DNA vaccine with dual CTLA-4/PD-1 immune checkpoint blockade in a murine melanoma model. To our knowledge, the adjuvant pIL12 has never been added to the combination of DNA vaccines and dual immune checkpoint blockade neither has been studied the involvement of tumor immunity in this particular combination. Due to many advantages, the skin was selected as a target tissue for DNA vaccination and MEA electrode designed for skin application was applied to promote intradermal gene electrotransfer of DNA vaccine. We confirmed the hypothesis that the intradermal DNA vaccination works hand in hand with dual CTLA-4 and PD-1 blockade, generating more robust antitumor activity compared to each treatment alone. These results indicate the potential of anticancer DNA vaccination and immune checkpoint blockade to enhance the efficacy of both treatment approaches and to overcome the limitations of single treatments.

## Supporting information

S1 Fig Tumor response in mice treated with pOVA DNA or pGP100 vaccine in combination with CTLA-4/PD-1 blockade. Changes in tumor size relative to the baseline measurement (naive mice) are presented.
(DOCX)

S2 Fig Flow cytometry analysis. Raw data of flow cytometry analysis, presenting the distribution of labeled spleen cells excised from treated mice. Cells included in presenting plots were double positive: CD3 (APC-Cy7) positive and CD4 (FITC) positive. Double positive cells were further divided to FoxP3 (APC) positive cells and FoxP3 (APC) negative cells.
(DOCX)

S3 Fig Immunohistochemical presentation of spleen samples. Histological observation of CD4^+^ and CD8^+^ T cells (in green) and FOXP3+ cells (in orange) in mouse spleens. DAPI was used to visualize cell nuclei (in blue). Scale bar: 500 μm.
(DOCX)

S4 Fig Immunohistochemical presentation of tumor samples. Histological observation of CD4^+^ and CD8^+^ T cells (in green) and FOXP3+ cells (in orange) in mouse tumors. DAPI was used to visualize cell nuclei (in blue). Scale bar: 500 μm.
(DOCX)
