# Supplementary figures and images for: Intradermal DNA vaccination combined with dual CTLA-4 and PD-1 blockade provides robust tumor immunity in murine melanoma
Source: PLoS One. 2019 May 31;14(5):e0217762. doi: 10.1371/journal.pone.0217762 (PMC6544376; doi:10.1371/journal.pone.0217762)

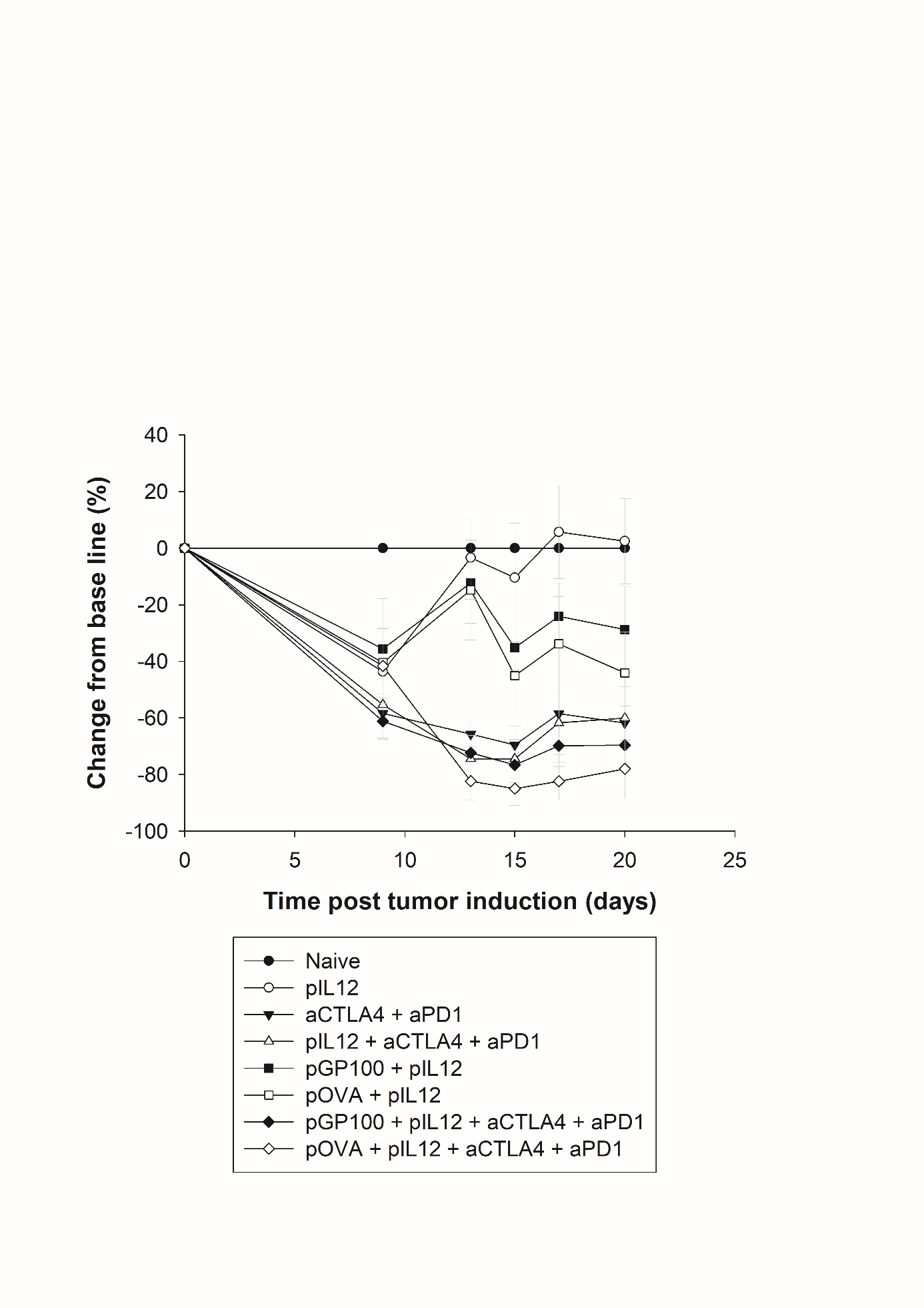


**S1 Fig**

Supplement: S1 Fig — Changes in tumor size relative to the baseline measurement (naive mice) are presented. (DOCX) [file pone.0217762.s001.docx]

**
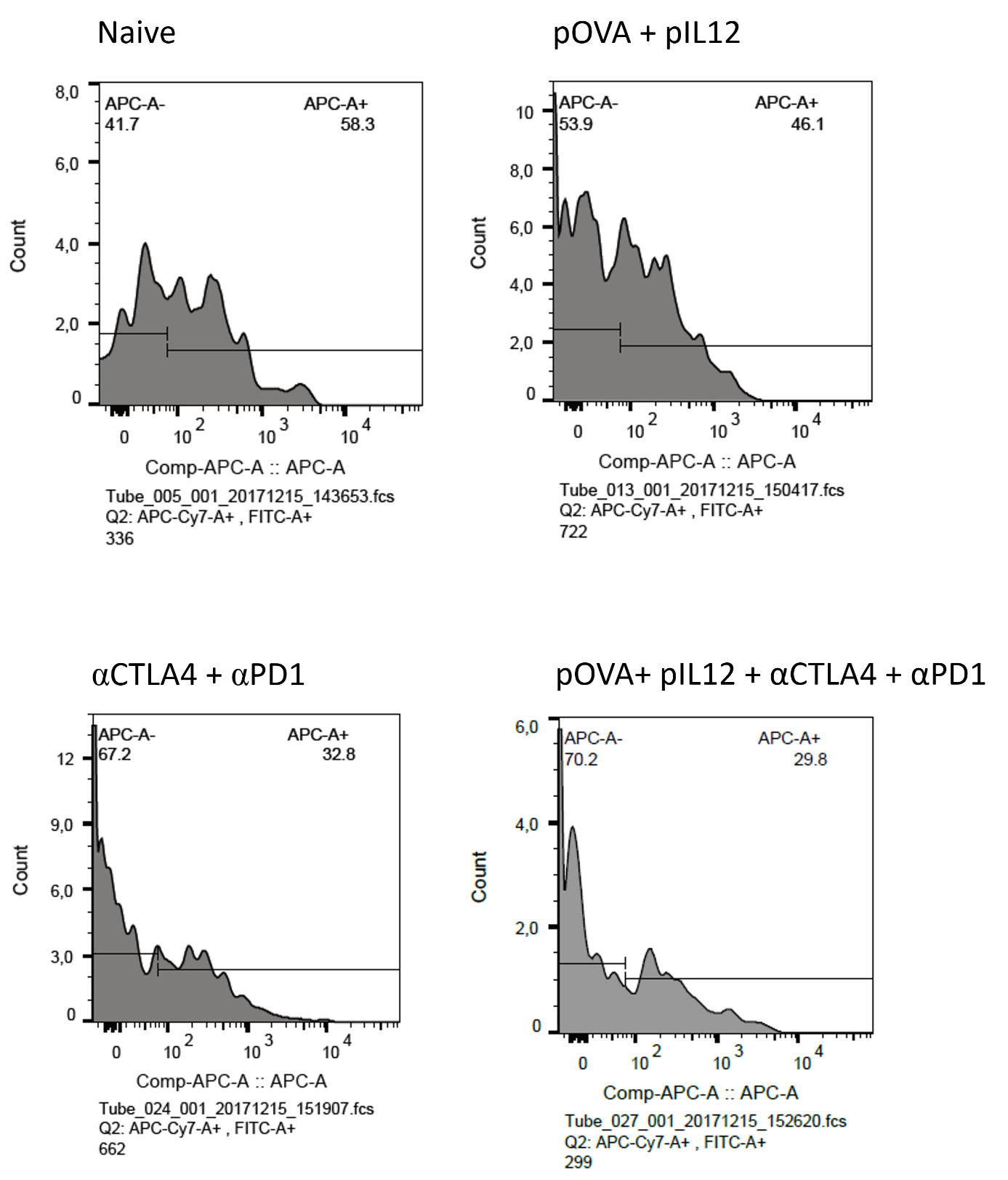
S2 Fig**

Supplement: S2 Fig — Raw data of flow cytometry analysis, presenting the distribution of labeled spleen cells excised from treated mice. Cells included in presenting plots were double positive: CD3 (APC-Cy7) positive and CD4 (FITC) positive. Double positive cells were further divided to FoxP3 (APC) positive cells and FoxP3 (APC) negative cells. (DOCX) [file pone.0217762.s002.docx]

**
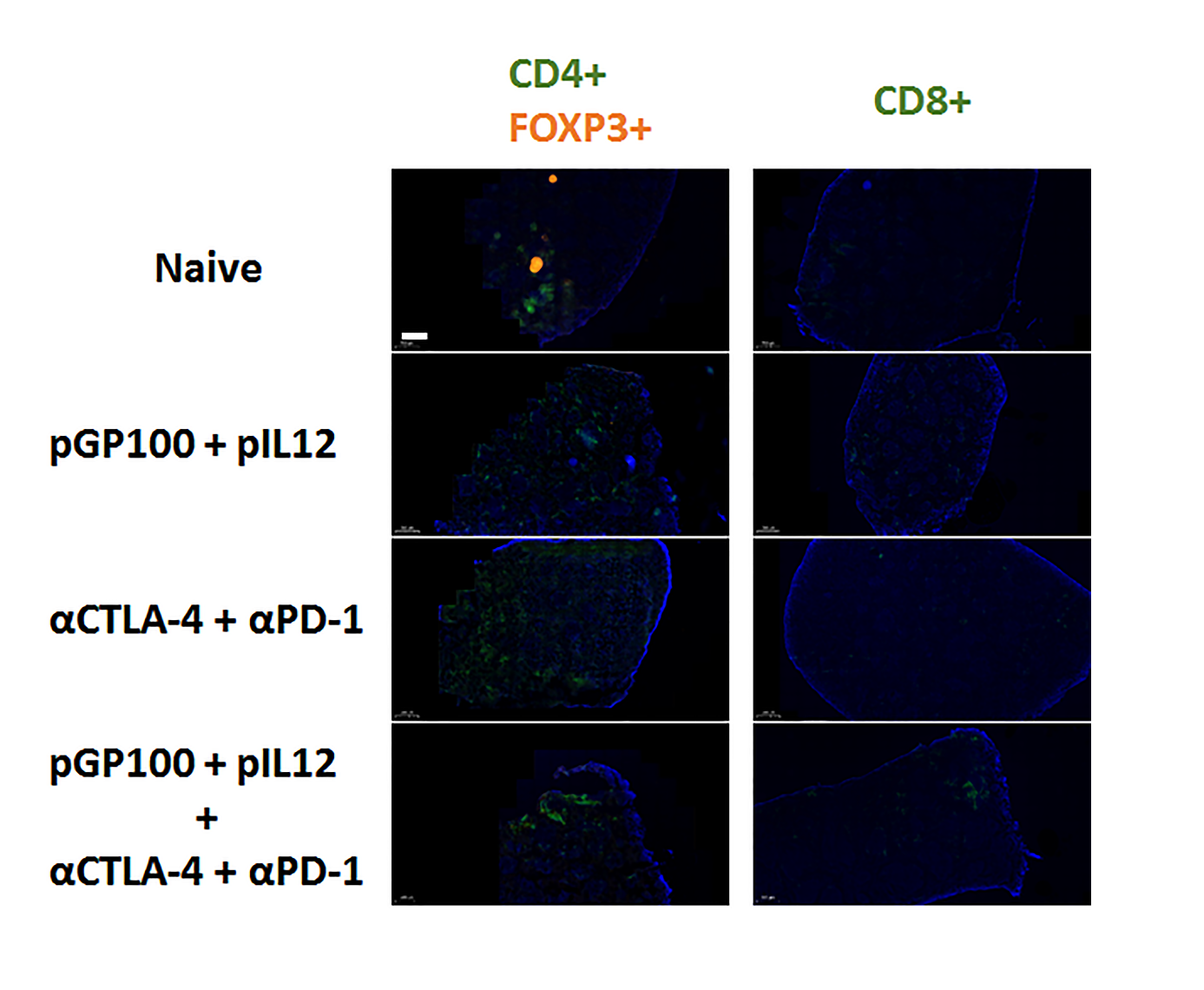
S3 Fig**

Supplement: S3 Fig — Histological observation of CD4+ and CD8+ T cells (in green) and FOXP3+ cells (in orange) in mouse spleens. DAPI was used to visualize cell nuclei (in blue). Scale bar: 500 μm. (DOCX) [file pone.0217762.s003.docx]

**
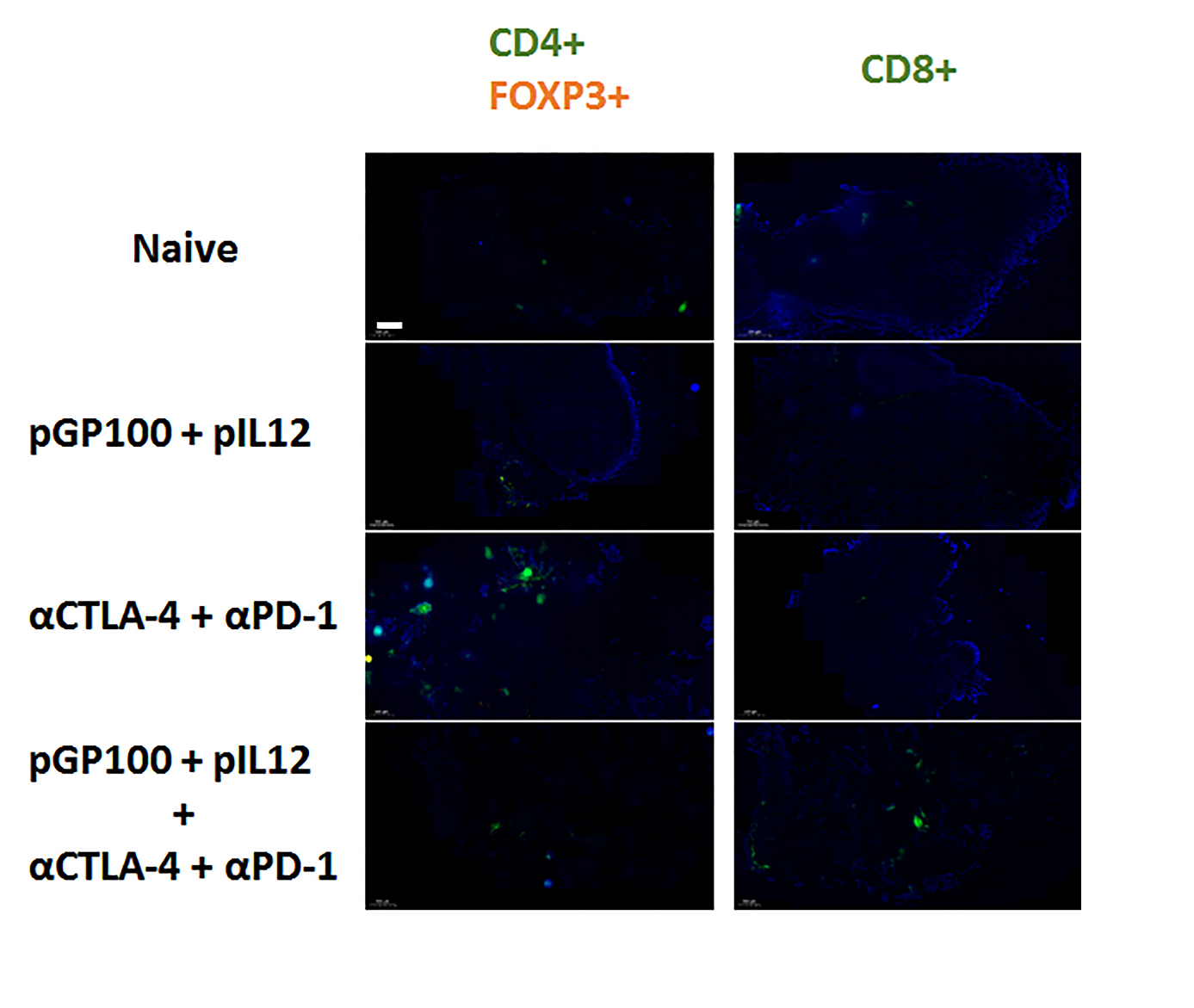
S4 Fig**

Supplement: S4 Fig — Histological observation of CD4+ and CD8+ T cells (in green) and FOXP3+ cells (in orange) in mouse tumors. DAPI was used to visualize cell nuclei (in blue). Scale bar: 500 μm. (DOCX) [file pone.0217762.s004.docx]
